# Breakfast habits and differences regarding abdominal obesity in a cross-sectional study in Spanish adults: The ANIBES study

**DOI:** 10.1371/journal.pone.0188828

**Published:** 2017-11-30

**Authors:** Beatriz Navia, Ana M. López-Sobaler, Tania Villalobos, Javier Aranceta-Bartrina, Ángel Gil, Marcela González-Gross, Lluis Serra-Majem, Gregorio Varela-Moreiras, Rosa M. Ortega

**Affiliations:** 1 VALORNUT Research Group, Department of Nutrition, Faculty of Pharmacy, Complutense University of Madrid, Madrid, Spain; 2 Department of Preventive Medicine and Public Health, University of Navarra, Pamplona, Spain; 3 CIBER: CB12/03/30038 Fisiopatología de la Obesidad y la Nutrición, CIBERobn, Instituto de Salud Carlos III (ISCIII), Madrid, Spain; 4 Department of Biochemistry and Molecular Biology II, Institute of Nutrition and Food Sciences, University of Granada, Granada, Spain; 5 ImFINE Research Group, Department of Health and Human Performance, Technical University of Madrid, Madrid, Spain; 6 Research Institute of Biomedical and Health Sciences & Medical School, University of Las Palmas de Gran Canaria, Las Palmas, Spain; 7 Department of Pharmaceutical and Health Sciences, Faculty of Pharmacy, CEU San Pablo University, Madrid, Spain; 8 Spanish Nutrition Foundation (FEN), Madrid, Spain; Fondazione Toscana Gabriele Monasterio, ITALY

## Abstract

**Background:**

Previous studies have indicated that breakfast has a protective effect against obesity. The aim of this study was to describe the breakfast habits of the Spanish adult population and to assess the possible association between breakfast frequency and the presence of abdominal obesity, in a cross-sectional analysis of the ANIBES Study.

**Methods:**

A representative sample of 1655 Spanish adults (aged 39±12 y; (mean±sd)) from the ANIBES Study was investigated. The final field work was carried out from mid-September to November (three months) 2013. Collected data included a dietary data collected by a 3-days food record, and health, socioeconomic, physical activity and anthropometric (weight, height and waist circumference) data. Abdominal obesity was defined as having a waist-to-height ratio ≥0.5. The adults were also classified into three groups based on the number of days they ate breakfast (never (0/3 days), sometimes (1-2/3 days) and always (3/3 days)). Logistic regression analyses were used to evaluate the association between breakfast and abdominal obesity.

**Results:**

In total, 3.6% of adults skipped breakfast and 14.1% ate breakfast sometimes. Having always breakfast was negatively associated with abdominal obesity [OR = 0.738 (0.558–0.975) *p* = 0.033]. The odds of abdominal obesity after full adjustment (age, gender, and educational and activity level) were 1.5 times higher for those who skipped breakfast when compared to those who always have breakfast. By correcting the model considered for other variables, the odds among smokers decreased when they have breakfast sometimes [OR = 0.032 (0.003–0.387) *p =* 0.007] and always [OR = 0.023 (0.002–0.270) *p =* 0.003] comparing with smokers who skip breakfast.

**Conclusion:**

Breakfast frequency could be negatively associated with abdominal obesity, especially among smokers.

## Introduction

Breakfast is considered the most important meal of the day [1]. Different studies have shown that eating breakfast improves daily food selection [2, 3], the intake of many nutrients [2, 3] and diet quality [1, 3, 4]. Regular breakfast consumption has also been associated with improved cardiovascular risk factors [5, 6] and weight/adiposity measures [4, 7–10].

Regarding obesity, previous research has shown that children and adolescents who eat breakfast regularly have a lower BMI and better long-term weight management than breakfast skippers [10]. In adults, it has also been observed that eating breakfast regularly decreased the prevalence of overweight and obesity [4, 7–9].

BMI is widely used to estimate the prevalence of obesity within a population and the risks associated with it; however, BMI does not account for the wide variation in body fat distribution [11] and individuals with excess fat in the intra-abdominal depots are at particular risk of the adverse health consequences [12–14].

However, there is limited information about the breakfast patterns of adults with abdominal obesity and how they may differ from their counterpart controls, as well as whether breakfast frequency influences abdominal obesity. In addition, most previous studies on breakfast habits have included children and adolescents [10, 15, 16], and data in the adult population [4, 17].

Therefore, the aim of the present study was to report the breakfast habits of a sample of the Spanish adult population and to assess the possible association between breakfast frequency and abdominal obesity as part of the ANIBES (Anthropometry, Intake, and Energy Balance in Spain) Study.

## Material and methods

The data used in this study were obtained as part of the ANIBES Study, a nationally representative survey conducted in the Spanish population aged 9–75 years; the design and methodology of the ANIBES Study have been described in detail elsewhere [18–22]. The final protocol was approved by the *Ethical Committee for Clinical Research of the Region of Madrid* (Spain). Written informed consent was obtained from all subjects. All data were collected by trained interviewers. Briefly, ANIBES study was performed to record food and beverage intake, dietary habits, and anthropometric data, as well as energy expenditure and physical activity patterns of the Spanish population. The target population consisted of all inhabitants living in Spain (excluding the autonomous cities of Melilla and Ceuta in the north of Africa), aged 9 to 75 years and living in municipalities of at least 2,000 inhabitants. The sample for the ANIBES Study was based on the 2012 census data published by the INE (Instituto Nacional de Estadística/Spanish Bureau of Statistics) for gender, age, habitat size and region. The total sample size was calculated based on a 0.05 probability of Type I error (rejecting a null hypothesis when it is true) and 0.1 probability of Type II error (accepting a null hypothesis when it is wrong) in the main outcome of the study (energy intake). No previous pre-recruitment was considered to minimize the risk of bias in responses. The final study sample included 2009 individuals aged 9 to 75 years. The present paper focused on the adult population (18–64 years, *n* = 1655). Data were collected between mid-September and mid-November 2013.

Survey component included a dietary study, data from the habitat, level of education, socioeconomic level, percentage of immigrant population, tobacco consumption, physical activity, and anthropometric measures.

### Dietary data

Dietary intake was assessed via face-to-face 24-hour recall (1-day intake, not included in the final data) and a 3-day food record using a tablet device (Samsung Galaxy Tab 27.0, Korea) on 2 weekdays and one weekend day, including information on all food and beverages consumed at home and away, as well as eating habits (e.g., recipes, brands, types of milk and fat spread usually consumed, among other data). Participants who declared or demonstrated that they were unable to use the tablet device were offered other options, such as using a digital camera and paper record and/or telephone interviews. A manual of procedures to facilitate food collection was provided to participants, in addition to a toll-free telephone number in case they had any questions regarding the software, use of the device, or food and beverage records. Food, beverages, and energy and nutrient intake were calculated using software (VD-FEN 2.1, Madrid, Spain) that was newly developed for the ANIBES study by the Spanish Nutrition Foundation and is based mainly on expanded and updated Spanish food composition tables [23].

Breakfast was defined by self-report and included consumption of any food or beverage at a meal occasion named by the respondent as breakfast. Subjects who consumed no food or beverages at breakfast, excluding water, coffee or other noncaloric beverages, were categorized as breakfast skippers [24]. We also calculated the energy provided by breakfast regarding total energy intake. An energy intake of 15–25% of total energy was considered adequate [24]. Participants were also asked about the time spent on breakfast, and the food variety at breakfast was calculated considering only the consumption of foods within the cereal, dairy, fruits and protein-rich food groups.

The adults were classified into three groups based on the number of days they ate breakfast: never (0/3 days), sometimes (1-2/3 days) and always (3/3 days).

### Anthropometric data

Weight, height, and waist circumference were measured using standardized procedures by well-trained interviewers to minimize the inter-observer coefficients of variation [25]. Weight was measured once with a Seca model 804 weighing scale (Medizinische Messsysteme und Waagen seit 1840, Hamburg, Germany; range 0.1–150 kg, precision 100 g). Height was assessed in triplicate using a Seca model 206 Stadiometer (range 70–205 cm, precision 1 mm). Waist circumference was measured in triplicate using a Seca 201 tape measure (Seca, Hamburg, Germany; range 0–150 cm, precision 1 mm). BMI was calculated as weight to height squared (kg/m^2^), and abdominal obesity was defined as having a waist to height ratio (WHtR) ≥0.5 in both men and women, as others authors have reported [14, 26, 27].

### Personal, socioeconomic and lifestyle data

A questionnaire was administered face-to-face in order to record the following data: age in completed years (which were later classified in 18–40/41–65 years), gender, habitant size (rural, semi-urban, or urban populations of 2,000–30,000, 30,000–200,000, and over 200,000 inhabitants, respectively), place of birth (no immigrant/immigrant), educational level according to years and type of education (primary or less/secondary/university). Smoking habits were grouped as smoker or non-smoker. Subjects were classified into two levels of physical activity based on two questions, one about the time spent in physical activity of daily life, and the second about time spent in structured physical exercise. “Active” subjects were those who performed at least 30 min per day of physical activity of daily life or at least 2 h per week of structured physical exercise; otherwise subjects were classified as “Inactive”.

### Statistical analysis

Data are presented as the means, standard deviations and percentages. Analyses were performed using SPSS version 22.0 (SPSS, Inc, Chicago, Illinois, USA). The Kolmogorov-Smirnoff test was used to test whether the variables followed a normal distribution to decide between a parametric or non-parametric analysis. Gender and abdominal obesity differences were assessed using T-test (normal distributions) or tests and Mann-Whitney test (non-normal distributions) tests. When comparing proportions, *z*-test was used. Kruskal-Wallis test was used to determine the differences between the three breakfast groups. Comparisons of the percentages of the categorized variables were conducted using Pearson’s Chi-square test. Backward stepwise logistic regression was conducted to examine the association of breakfast frequency and abdominal obesity, after controlling for confounders (gender, age, education level, smoking and physical activity level). The dependent variable was WHtR. Reference groups comprised individuals without abdominal adiposity (WHtR<0·5). Logistic regression analysis results from the reduced model were expressed as odds ratios (OR) and their respective 95% CI. We used the Hosmer-Lemeshow test to determine the model fit *p<*0.05 was considered statistically significant.

## Results

Personal, anthropometric, sociodemographic and lifestyle factors of the studied sample have been described previously [21, 22, 28] but are presented again for simple characterization (Table 1). Some 58.4% of the population had abdominal obesity and the percentage is significantly higher in men. Most participants had a high school diploma (48.9%). Approximately a third were smokers and two thirds were physicaly inactive.

**Table 1:** Characteristics of the study population.

	Total	Men	Women
n	1655	798	857
Age (y) (m±sd)	40.0±12.2	39.6±12.2	40.3±12.2
18–40 (%)	53.4	54.5	52.3
41–64 (%)	46.6	45.5	47.7
Weight (kg) (m±sd)	74.2±16.48	82.4±15.34 *	66.6±13.62 *
Height (cm) (m±sd)	167.7±9.35	174.5±6.95 *	161.3±6.37 *
BMI (kg/m^2^) (m±sd)	26.3±5.15	27.1±4.87 *	25.6±5.3 *
Waist circumference (cm) (m±sd)	88.1±14.5	93.8±13.61 *	82.7±13.19 *
WHtR (m±sd)	0.53±0.08	0.54±0.08 *	0.51±0.09 *
WHtR >0.5 (%)	58.4	64.7 *	52.5 *
**Habitat size**^**a**^ **(%)**			
Rural	34.1	33.3	34.8
Semi-urban	33.9	35.6	32.3
Urban	32.0	31.0	32.9
Level of education (%)			
Primary or less	26.8	26.6	27.0
Secondary	48.9	49.6	48.3
University	24.3	23.8	24.7
Immigrant population (%)	6.4	7.3	5.6
Smoking status (%)			
Non smoker	65.0	60.2 *	69.5 *
Smoker	35.0	39.8 *	30.5 *
Physical Activity level (%)			
Active	32.6	39.8 *	25.8 *
Inactive	67.4	60.2 *	74.2 *

m: mean; sd: standard deviation; BMI: Body mass index; WHtR: Waist to height ratio

(a) Habitat size: rural populations: 2,000–30,000; semi-urban: 30,000–200,000; urban population: over 200,000 inhabitants.

Student’s t test for normally distributed variables and Mann Withney test for variables with non-normal distribution. Z test proportions for categorical variables.

(*) Significant differences regarding gender.

In total, 3.6% of the study population skipped breakfast, including significantly more men than women (Table 2); 2% did not eat anything for breakfast, and 1.6% had only coffee or tea for breakfast (Table 3). Skipping breakfast occurred more frequently in young adults (5.8% in 18–29 y versus 1.2% in 60–64 y, p<0.05), in males (4.8% versus 2.5% in females, p<0.05), in rural populations (5.2% versus 1.6% in semiurban and 4.0% in urban, p<0.05), in immigrant populations (7.6% versus 3.4%, p<0.05), in smokers (5.3% versus 2.7%, p<0.05) and in those less physically active (6.9% versus 2.7%, p<0.05). Breakfast represented 16.7% of total daily energy intake, and intake from breakfast was inadequate in 59.2% of the adults, being more frequently insufficient (<15%) than excessive (>25%) (Table 2).

**Table 2:** Breakfast habits in the study population.

	Total	Men	Women
n	1655	798	857
Age (y) (m±sd)	40.0±12.2	39.6±12.2	40.3±12.2
18–40 (%)	53.4	54.5	52.3
41–64 (%)	46.6	45.5	47.7
Breakfast (%):			
Never	3.6	4.8*	2.5*
Sometimes	14.1	16.9*	11.6*
Always	82.3	78.3*	86.0*
Total energy intake (kcal/day) (m±sd)	1.816±512	1.966±543***	1.675±437***
(MJ/day) (m±sd)	7.6±2.1	8.2±2.3***	7.0±1.8***
Breakfast energy intake (kcal/day) (m±sd)	301.5±164.3	313.5±178.3**	290.5±149.7**
(MJ/day) (m±sd)	1.3±0.7	1.3±0.8**	1.2±0.6**
Breakfast energy intake (% total energy intake) (m±sd)	16.7±8.3	15.94±8.3***	17.4±8.2***
Individuals whose breakfast provides: (%)			
15–25% total energy intake	40.8	39.0	42.5
<15% total energy intake	44.0	47.9*	40.4*
>25% total energy intake	15.2	13.0*	17.1*
Food variety at breakfast (number of foods at breakfast/day) (m±sd)	2.20±0.94	2.21±1.01*	2.19±0.87*
Time used for breakfast (minutes/day) (m±sd)	12.48±8.86	11.94±9.39*	12.96±8.33*

m: mean; sd: standard deviation; Student’s t test for normally distributed variables and Mann Withney test for variables with non-normal distribution. Z test proportions for categorical variables.

* *p<*0.05

** *p<*0.01

*** *p<*0.001.

**Table 3:** More frequently consumed breakfasts (% consumers).

	Total	Men	Women
Only coffee or tea	1.6	1.8	1.4
Only dairy products	10.0	10.6	9.6
Only cereals	0.4	0.1	0.6
Only fruit	1.1	1.7*	0.6*
Dairy products + Cereals	34.6	34.9	34.4
Dairy products + Fruit	1.3	1.6	1.1
Cereals + Fruit	1.4	1.7	1.1
Dairy products + Cereals + Fruit	16.8	12.4*	20.8*
Protein-rich foods + Cereals + Dairy products	8.0	8.1	7.8
Dairy products + Cereals + Fruit + Protein-rich foods	10.4	11.0	9.8
Other breakfasts	14.5	16.2	13.4
None of the above	5.5	6.5	4.6

Z test proportions.

* *p<*0.05.

The most frequently consumed food groups at breakfast were dairy products with cereals (34.6%); dairy products with cereals and fruit (16.8%) and dairy products with cereals, fruit and protein-rich foods (10.4%), whereas 10% of adults only had some dairy product for breakfast (Table 3).

The most consumed food groups were dairy products (88.9%) (Table 4), especially semi-skimmed milk (43.8%) and less frequently whole milk (28.2%) and skimmed milk (22.3%). There were more men who consumed whole milk at breakfast than women (*p<*0.05), whereas there were more women who chose skimmed milk and soya drinks more often than men (*p<*0.05) (S1 Table).

**Table 4:** Food groups consumed at breakfast (% consumers).

	Total	Men	Women
Dairy products	88.9	86.7*	90.9*
Cereals	84.2	81.7*	86.4*
Fruit + Juice	38.8	37.6	40.0
Protein-rich foods	26.3	28.8*	24.1*
Other foods	5.8	6.0	5.6
Beverages	8.3	6*	10.5*
Fats	43.3	38.7*	47.6*
Other products	88.6	85.5*	91.5*

Z test proportions.

* *p<*0.05.

In the study population, cereals were the second most chose food group at breakfast (84.2%), in particular bread (51.6%), and in lower proportions, cookies (25.7%), cakes and pastries (20.6%), and Ready-to-eat-cereals (RTEC) (12.9%). Furthermore, 38.8% of the population included fruit or fruit juice, and 26.3% included protein-rich foods, especially charcuterie and other meat products (20%) and eggs (7.2%). Regarding to fats, 29.2% included oils, mainly olive oil (25.3%), while 22.9% used other fats (butter, margarine, etc.). Butter, margarine and other solid fats were also significantly more consumed by women than men, similar to jam consumption. Sugar was the most widely used sweetener (46.9%), with no gender differences, although saccharin was more frequently used in women than men (*p<*0.05) (S1 Table). In food variety at breakfast, we just have been taking into account foods that belong to one of this food groups: cereals, dairy, fruits and protein-rich foods. The variety at breakfast was 2.20±0.94 foods per day and was significantly higher in men than in women; however, women spent more time on breakfast than men (*p<*0.05) (Table 2).

The prevalence of breakfast consumption among adults with abdominal obesity compared to those without abdominal obesity is presented in Table 5. We found no difference in the prevalence of breakfast skippers regarding WHtR categories (Table 5), but men who never had breakfast had a higher waist circumference and WHtR than men who sometimes ate breakfast (Table 6).

**Table 5:** Breakfast habits regarding WHtR categories.

	WHtR < 0.5			WHtR ≥ 0.5		
	Total	Men	Women	Total	Men	Women
N	689	282	407	966	516	450
Age (y) (m±sd)	34.0±11.0***	32.5±11.2***	35.0±10.7***	44.2±11.2***	43.5±10.9***	45.0±11.5***
18–40 (%)	73.4*	78.4*	70*	39*	41.5*	36.2*
41–64 (%)	26.6*	21.6*	30*	61*	58.5*	63.8*
Breakfast (%):						
Never	2.9	3.6	2.5	4.1	5.5	2.4
Sometimes	16.4*	20.9*	13.3	12.5*	14.7*	10
Always	80.7	75.5	84.3	83.4	79.8	87.5
Total energy intake (kcal/day) (m±sd)	1886±543***	2102±581***	1736±460***	1765±482***	1892±507***	1620±407***
(MJ/day) (m±sd)	7.9±2.3***	8.8±2.4***	7.3±1.9***	7.4±2.0***	7.9±2.1***	6.8±1.7***
Breakfast energy intake (kcal/day) (m±sd)	310.8±169.3	339.3±189.0**	291.2±151.6	294.9±169.3	299.4±170.8**	289.9±148.1
(MJ/day) (m±sd)	1.3±0.7	1.4±0.8**	1.2±0.6	1.2±0.7	1.3±0.7**	1.2±0.6
Breakfast energy intake (% total energy intake) (m±sd)	16.6±8.0	16.1±8.0	16.8±8.5*	16.8±8.5	15.8±8.5	18.0±8.3*
Individuals whose breakfast provides: (%)						
15–25% energy total	39.3	36.3	41.4	41.9	40.5	43.4
<15% energy total	45.7	49.5	43.1	42.8	47.1	38.1
> 25% de la energy total	15	14.3	15.5	15.3	12.4	18.5
Food variety at breakfast (number of foods at breakfast/day) (m±sd)	2.25±1.00*	2.33±1.12*	2.20±0.91	2.16±0.89*	2.13±0.93*	2.18±0.84
Time used for breakfast (minutes/day) (m±sd)	12.12±8.72	11.72±9.75	12.40±7.95	12.73±8.96	12.07±9.19	13.47±8.63

m: mean; sd: standard deviation; Student’s t test for normally distributed variables and Mann Whitney test for variables with non-normal distribution. Z test proportions for variables expressed in proportions. Significant differences between adults with WHtR < 0.5 and WHtR ≥ 0.5.

* *p<*0.05

** *p<*0.01

*** *p<*0.001.

**Table 6:** Anthropometric parameters regarding the pattern of breakfast intake.

	Never			Sometimes			Always		
	Total	Men	Women	Total	Men	Women	Total	Men	Women
Age (y) (m±sd)	37.1±11.7 b	37.6±12.8	36.2±12.2 b	35.4±12.9 c	36.2±12.8 c	34.3±13.0 c	40.9±11.9 b,c	40.5±12.0 c	41.2±11.9 b,c
Weight (kg) (m±sd)	76.2±17.4	83.6±16.5	62.7±9.2	74.1±16.6	81.1±15.4	64.7±13.3	74.0±16.4	82.5±15.4	66.9±13.7
Height (cm) (m±sd)	169.4±9.8	174.3±7.0	160.6±7.8	169.7±9.5 c	174.7±6.8	160.6±6.0	167.4±9.3 c	174.5±7.0	161.5±6.4
BMI (kg/m^2^) (m±sd)	26.5±5.2	27.6±5.6	24.4±3.8	25.9±4.9	26.6±4.8	25.1±5.1	26.4±5.2	27.1±4.9	25.7±5.4
Waist circumference (cm) (m±sd)	90.3±15.2	96.2±14.1 a	79.4±10.6	86.8±14.2	91.5±13.7 a,c	80.3±12.2	88.1±14.5	94.1±13.6 c	83.1±13.3
WHtR (m±sd)	0.53±0.08	0.55±0.08 a	0.50±0.07	0.51±0.08	0.52±0.08 a,c	0.50±0.08	0.53±0.09	0.54±0.08 c	0.52±0.09

m: mean; sd: standard deviation; WHtR: Waist to height ratio; Kruskal Wallis Test. The differences are according to breakfast frequency

a: differences between “never” vs “sometimes”

b: differences between “never” vs “always”

c: differences between “sometimes” vs “always”.

*p<*0.05.

In food variety at breakfast, we just have been taking into account foods that belong to one of this food groups: cereals, dairy, fruits and protein-rich foods.

An inverse association was observed between breakfast frequency and abdominal obesity by Chi-square test (*p =* 0.046). Binary logistic regression analysis also showed that breakfast was negatively associated with abdominal obesity (Table 7). After adjusting for age, the odds of abdominal obesity decreased in those who ate breakfast sometimes and always, compared to those who skipped breakfast. When adjusting also by other variables (gender, smoking, educational level and physical activity), the odds of abdominal obesity decreased only in those who always ate breakfast. There was a significant interaction between breakfast frequency and smoking, and the odds of abdominal obesity (adjusted by age, gender, educational level and physical activity) decreased among smokers when they ate breakfast sometimes [OR = 0.032 (0.003–0.387) *p* = 0.007] and always [OR = 0.023 (0.002–0.270) *p* = 0.003] comparing with smokers who skipped breakfast.

**Table 7:** Association of breakfast frequency and abdominal obesity in Spanish adults. Logistic regression analysis.

	OR (95%CI)	p	OR^1^	p	OR^2^	p
Never	1		1		1	
Sometimes	1.355 (0.782–2.349)	0.279	0.145 (0.030–0.710)	0.017	1.397 (0.704–2.771)	0.338
Always	0.738 (0.558–0.975)	0.033	0.122 (0.026–0.576)	0.008	0.662 (0.464–0.946)	0.024

OR^1^: Odds ratio adjusted for age

OR^2^: Odds ratio adjusted for age, gender, smoking, educational level and activity.

We have also found significant differences in breakfast composition regarding abdominal obesity (Table 8). Comparing with adults without abdominal obesity, there were more consumers of coffee, tea, saccharin (men and women) and skimmed milk (women) with abdominal obesity, while there were less consumers of cocoa, chocolate and juice (men and women), cakes and pastries, RTEC, fruit and eggs (men). (S2 Table). We did not observe differences in the type of breakfast regarding WHtR categories (Table 9) but, for both total sample and males, the variety of foods at breakfast was lower among those with abdominal obesity (Table 5).

**Table 8:** Foods consumed at breakfast regarding WHtR categories (% consumers).

	WHtR < 0.5			WHtR ≥ 0.5		
	Total	Men	Women	Total	Men	Women
Dairy products	89.9	88.6	90.7	88.2	85.6	91.1
Cereals	84.1	83.2	84.7	84.2	80.8	88.0
Fruit + Juice	41.8*	42.9*	41.1	36.7*	34.7*	39.0
Protein-rich foods	26.2	30.0	23.6	26.4	28.1	24.5
Other foods	5.7	7.4	4.4	5.9	5.2	6.7
Beverages	9.4	6.0	11.8	7.6	6.0	9.3
Fats	40.9	37.2	43.5	45.0	39.5	51.3
Other products	86.8	84.0	88.7	89.9	86.2	94.0

Z test proportions. The differences are between groups of the same gender.

* *p<*0.05

**Table 9:** More frequently consumed breakfasts regarding WHtR categories (% consumers).

	WHtR < 0.5			WHtR ≥ 0.5		
	Total	Men	Women	Total	Men	Women
Only coffee and tea	0.4	0.4	0.5	2.4	2.6	2.2
Only dairy products	11.2	11.0	11.3	9.3	10.4	8.0
Only cereals	0.6	0	1.0	0.2	0.2	0.2
Only fruit	1.3	1.8	1.0	0.9	1.6	0.2
Dairy products + Cereals	33.3	34.1	32.8	35.6	35.3	35.9
Dairy products + Fruit	1.3	1.5	1.3	1.3	1.6	0.9
Cereals + Fruit	1.8	2.6	1.3	1.1	1.2	0.9
Dairy products + Cereals + Fruit	18.0	13.2	21.3	15.9	12.0	20.3
Protein-rich foods + Cereals + Dairy products	7.6	7.3	7.8	8.2	8.6	7.8
Dairy products + Cereals + Fruit + Protein-rich foods	10.4	12.1	9.3	10.3	10.4	10.2
Other breakfasts	9.1	11.4	7.5	8.9	8.8	9.1
None of the above	4.9	4.8	5.0	5.9	7.4	4.2

Finally, in our study we found 16.9% of regular exercises in the group who did not have breakfast versus 33.9% (*p<*0.05) and 33.1% (*p<*0.05) in the groups who ate breakfast sometimes and always, respectively. Moreover, we also found that 6.9% of inactive men skipped breakfast while do it 3.5% of active men (*p<*0.05).

## Discussion

In several studies, breakfast has been considered the most important meal of the day, in part because of its nutritional benefits, as well as its associations with other positive outcomes [29]. In the present study, 82.3% of the sample reported consuming breakfast daily, whereas 14.1% did not always have breakfast, and 3.6% (more men than women) skipped this meal.

Awareness campaigns conducted in Spain about the importance of improving breakfast habits [30–33] may be the cause of the lower percentage of individuals who skipped their first meal of the day, compared with the results of other studies. Specifically, Reeves et al. [34] in a representative sample of the UK adult population (≥18 years), found that approximately 6% of the sample never had breakfast. Chung et al. [35] showed that of a sample of 11,801 Korean adults aged 24–60 years, 21,6% skipped breakfast with the highest skipping rate in those aged 20–34 years.

However, as other authors have indicated, differences in socioeconomic status, gender, ethnicity and cultural norms could also explain the variations found in the data obtained from different studies [34]. Furthermore, according to O’Neil et al. [24], breakfast, breakfast consumption, and breakfast skipping are defined differently across research studies, and the lack of a standard definition of breakfast makes it difficult the comparisons between them. Some of the criteria applied to define breakfast are based on weekly frequency, time of day, timing in relation to waking and daily activities, types of food or beverage consumed, and amount of energy provided by this meal. In this regard, and in light of the criterion we have used to define breakfast, although in our study only 3.6% of the adults reported that they did not eat breakfast, 44% ate less than 15% of their calories in this first meal of the day, which may be considered an insufficient breakfast.

Factors contributing to skipping breakfast include a lack of knowledge about the relationship between nutrition and health [36] and lack of time to make or prepare breakfast [37], especially among adolescent girls [38], and concern about body weight [37, 38]. However, the last educational campaigns carried out to promote an adequate breakfast may have modified this last factor since several studies have indicated that breakfast consumers believe that breakfast helps with weight control [34, 39]. This could also explain the higher percentage of consumers of breakfast observed in women, as they have a greater concern about body weight than men [40].

O’Neil et al. [24] have also indicated that children tend to eat breakfast less often as they age, so adolescents and young adults typically have the lowest rates of breakfast consumption, and older adults typically have the highest rates of breakfast consumption. In the present study, in line with these previous findings and those of other studies conducted in adults [8, 35], omitting breakfast was highest among those 18 to 29 years (5.8%) and lowest among those 60 to 64 years (1.2%).

The breakfast most frequently consumed in Spain consisted of a dairy product, particularly skimmed milk, and cereal, especially bread, which differs from the findings in other populations. Reeves et al. [34] found that the most commonly consumed foods for breakfast were cereal, bread or toast, and porridge or granola as well as tea and coffee. In the American NHANES III, cereals were also the most popular breakfast choice [7]. We also found differences between sexes, and as other studies indicated, the consumers of low calorie sweeteners were more likely to be women [41].

On the other hand, the percentage of breakfast consumers in our study who reported smoking (43.3% and 32.6% in adults who ate breakfast “sometimes” and “always”, respectively) was lower than the proportion of smokers in the breakfast nonconsumers group (50.8%) (p<0.05). This is in agreement with other studies that had reported that breakfast skippers are more likely to have unhealthier habits, such smoking, than breakfast consumers [8, 42]. In fact, it has been suggested that breakfast consumption may serve as a marker for a healthier lifestyle [34, 43].

In contrast with other studies conducted in Spain [44], Sweden [9] and Taiwan [45], but in agreement with the results obtained in Canadian adults [42] and in studies conducted in the United States [8], breakfast consumption (versus non-consumption) was not associated with BMI or the prevalence of overweight/obesity. However, the results of the present study showed an unfavourable association between breakfast consumption and abdominal obesity in Spanish adults. Moreover, men who usually skipped breakfast had a higher waist circumference and higher WHtR than men who usually had breakfast.

Our results are supported by the findings of several studies that showed detrimental effects of skipping breakfast on abdominal obesity. Azadbakht et al. [4] showed a smaller percentage of breakfast consumers in the third tertile of waist circumference in young Isfahanian women. Deshmukh-Taskar et al. [46] found in a study of 5316 young adults (20–39 years of age) that the mean waist circumference was lower in breakfast consumers than in breakfast skippers, independent of the type of breakfast consumed. Similarly, data from an Australian longitudinal study showed that skipping rather than eating breakfast over a long period of time resulted in detrimental effects on cardiometabolic risk profile, including a higher waist circumference [47]. All these results may have important implications, as a combination of obesity and abdominal obesity, or abdominal obesity by itself, has been found to be associated with obesity-related metabolic disorders and all-cause mortality in adults [48–50].

On the other hand, it is important to note that in our study, smokers who eat breakfast sometimes or always, had a lower odd of abdominal obesity than smokers who skipped breakfast. Smoking by itself is a risk factor for disease [51] and thus breakfast could especially benefit this particular population group.

The factors associated with skipping breakfast and increased adiposity are unclear; however, there are several potential explanations for this finding. Several authors report that daily breakfast eaters seem to be more physically active than breakfast skippers [8, 52]. In our study, we found fewer physically active subjects in the group who never had breakfast than in those who sometimes or always ate breakfast, and there were more inactive men who skipped breakfast compared with active men.

In fact, there appears to be a greater likelihood of health-compromising behaviours in people who miss breakfast, including increased prevalence of smoking, poorer food choices and greater energy intake from snacks [53]. On the other hand, it should be noted that breakfast type could be an important factor which modulates the risk of having abdominal obesity [37, 54].

Regarding the types of dietary groups consumed at breakfast, to our knowledge, there are no reports on the breakfast food group intakes in adults with abdominal obesity versus non-abdominal obesity. In our study we found that there are fewer consumers of cocoa, chocolates, juices, cakes and pastries and eggs and more consumers of skimmed milk and saccharin in those groups with higher WHtR (≥0.5). However, their breakfast had also less food variety, and there were fewer consumers of fresh fruit and RTEC, and thus an increase in food variety and a higher consumption of these foods at breakfast could have positive effects on abdominal obesity.

Our results should be interpreted with caution due to some limitations. This was a cross-sectional study. Therefore, the associations reported cannot be identified and/or can only be interpreted as hypothetical causal relations. The frequency of breakfast has been established based on a 3-day meal record and it is possible that some subjects have not been correctly identified as "breakfast skippers" or "always have breakfast". Another limitation of our study is a possible dietary underreporting that could affect to the energy and foods reported at breakfast. Nevertheless, the frequency of breakfast was established based on the self-declaration of the participant, and does not depend on the energy declared. On the other hand, ANIBES data were representative of the Spanish population, and height, weight and waist were measured, not self-reported, which is a more accurate assessment procedure that strengthens the data.

In conclusion, although the majority of Spanish adults regularly ate breakfast, 3.6% of the adults skipped breakfast. Omitting breakfast was associated with other unhealthy habits, such as smoking and being less active. Regular breakfast consumption was negatively associated with abdominal obesity in the Spanish adult population. Furthermore, men with abdominal obesity had less food variety at breakfast and lower consumption of fruit in their first meal of the day comparing with those with no abdominal obesity. These results reinforce the importance of nutritional education in the Spanish adult population to promote the importance of an adequate breakfast in preventing abdominal obesity.

## Supporting information

S1 Table Foods consumed at breakfast (% consumers). Z test proportions.* *p<*0.05.
(DOCX)

S2 Table Foods consumed at breakfast (% consumers). **Differences regarding WHtR categories.** Z test proportions. The differences are between the same gender groups.* *p<*0.05.
(DOCX)
