# Correction: Breakfast habits and differences regarding abdominal obesity in a cross-sectional study in Spanish adults: The ANIBES study

**DOI:** 10.1371/journal.pone.0203341

**Published:** 2018-08-28

**Authors:** Beatriz Navia, Ana M. López-Sobaler, Tania Villalobos, Javier Aranceta-Bartrina, Ángel Gil, Marcela González-Gross, Lluis Serra-Majem, Gregorio Varela-Moreiras, Rosa M. Ortega

There is an error in the data presentation of Supporting Information file S2 Table. Please view the correct S2 Table below.

## Supporting information

S2 Table Foods consumed at breakfast (% consumers). **Differences regarding WHtR categories.** Z test proportions. The differences are between the same gender groups.* *p*<0.05.
(DOCX)
