# Supplementary material for: Breakfast habits and differences regarding abdominal obesity in a cross-sectional study in Spanish adults: The ANIBES study
Source: PLoS One. 2017 Nov 30;12(11):e0188828. doi: 10.1371/journal.pone.0188828 (PMC5708749; doi:10.1371/journal.pone.0188828)
Supplement: S1 Table — Z test proportions.* p<0.05. (DOCX) [file pone.0188828.s001.docx]

**S1 Table. Foods consumed at breakfast (% consumers).**

|  | **Total** | **Men** | **Women** |
| --- | --- | --- | --- |
| **Dairy products** | **88.9** | **86.7*** | **90.9*** |
| Semi-skimmed cow milk | 43.8 | 42.6 | 44.9 |
| Whole cow milk | 28.2 | 31.9* | 24.9* |
| Skimmed cow milk | 22.3 | 19.3* | 25.0* |
| Other milks | 1.7 | 1.7 | 1.7 |
| Yoghurt and fermented milks | 8.1 | 10.1* | 6.4* |
| Cheese | 11.2 | 11.2 | 11.1 |
| Other dairy products (custard, curd, etc.) | 3.1 | 3.4 | 2.8 |
| **Cereals** | **84.2** | **81.7*** | **86.4*** |
| Bread | 51.6 | 47.2* | 55.7* |
| Ready-to-eat-cereals (RTEC) | 12.9 | 10.7* | 15.0* |
| Biscuits | 25.7 | 23.4* | 27.8* |
| Muffin | 10.0 | 11.6* | 8.6* |
| Cakes and pastries | 20.6 | 20.9 | 20.3 |
| Grains and flours | 8.1 | 7.1 | 9.1 |
| Fruit + Juice | 38.8 | 37.6 | 40.0 |
| Fresh fruit | 17.5 | 16.3 | 18.6 |
| Fruit nectar | 1.0 | 1.2 | 0.9 |
| Juices | 18.3 | 19.9 | 16.9 |
| Tomato | 11.0 | 9.3* | 12.6* |
| **Protein-rich foods** | **26.3** | **28.8*** | **24.1*** |
| Charcuterie and other meat products (chorizo, bacon, ham, sausages, etc) | 20.0 | 21.4 | 18.6 |
| Eggs | 7.2 | 9.0* | 5.5* |
| Meats | 1.5 | 1.8 | 1.2 |
| Fishes | 1.9 | 1.9 | 1.9 |
| **Other foods** | **5.8** | **6.0** | **5.6** |
| Greens and vegetables | 4.7 | 4.8 | 4.6 |
| Legumes | 0.4 | 0.5 | 0.2 |
| Pasta | 0 | 0 | 0 |
| Appetizers (crackers, chip potatoes, corn snacks, etc.) | 1 | 1 | 1 |
| Precooked foods (ready-to-eat creams and soups, croquettes, etc) | 1 | 1 | 1 |
| **Beverages** | **8.3** | **6*** | **10.5*** |
| Soya drinks | 4.9 | 2.2* | 7.4* |
| Sugared soft drinks | 1.8 | 2.3 | 1.3 |
| Sugar free soft drinks | 0.9 | 0.5 | 1.2 |
| Sports drinks | 0.1 | 0.1 | 0.1 |
| Other nonalcoholic beverages | 0.1 | 0.1 | 0.1 |
| Alcoholic beverages high alcohol (gin, whisky,etc) | 0.7 | 0.5 | 0.8 |
| Low-grade alcoholic beverages (wine, beer, cava, cider, etc) | 0.2 | 0.4 | 0.1 |
| **Fats** | **43.3** | **38.7*** | **47.6*** |
| Olive oil | 25.3 | 24.8 | 25.7 |
| Butter, margarine and other solid fats | 22.9 | 17.8* | 27.5* |
| Other oils | 3.9 | 4.0 | 3.8 |
| **Other products** | **88.6** | **85.5*** | **91.5*** |
| Cocoa | 23.1 | 24.5 | 21.8 |
| Sugars | 46.9 | 46.9 | 46.8 |
| Jams and others | 13.1 | 10.3* | 15.6* |
| Other chocolates | 7.9 | 8.7 | 7.2 |
| Saccharin | 13.2 | 10.1* | 16.0* |
| Sauces and condiments | 12.0 | 12.0 | 12.0 |
| Supplements and meal substitutes | 1.7 | 1.7 | 1.8 |

Z test proportions.* *p<*0.05.
