# Supplementary material for: Breakfast habits and differences regarding abdominal obesity in a cross-sectional study in Spanish adults: The ANIBES study
Source: PLoS One. 2017 Nov 30;12(11):e0188828. doi: 10.1371/journal.pone.0188828 (PMC5708749; doi:10.1371/journal.pone.0188828)
Supplement: S2 Table — Differences regarding WHtR categories. Z test proportions. The differences are between the same gender groups.* p<0.05. (DOCX) [file pone.0188828.s002.docx]

**S2 Table. Foods consumed at breakfast (% consumers). Differences regarding WHtR categories.**

|  | **WHtR < 0.5** | | | **WHtR ≥ 0.5** | | |
| --- | --- | --- | --- | --- | --- | --- |
|  | **Total** | **Men** | **Women** | **Total** | **Men** | **Women** |
| **Dairy products** | **89.9** | **88.6** | **90.7** | **88.2** | **85.6** | **91.1** |
| Semi-skimmed cow milk | 43.5 | 42.5 | 44.1 | 44.1 | 42.7 | 45.7 |
| Whole cow milk | 30.7 | 36.3 | 26.8 | 26.5 | 29.5 | 23.2 |
| Skimmed cow milk | 19.2* | 15.8 | 21.6* | 24.4* | 21.2 | 28.1* |
| Other milks | 1.8 | 1.5 | 2.0 | 1.6 | 1.8 | 1.3 |
| Yoghurt and fermented milks | 8.8 | 12.5 | 6.3 | 7.7 | 8.8 | 6.5 |
| Cheese | 12.5 | 13.6 | 11.8 | 10.2 | 10.0 | 10.5 |
| Other dairy products (custard, curd, etc.) | 3.6 | 4.4 | 3.0 | 2.7 | 2.8 | 2.7 |
| **Cereals** | **84.1** | **83.2** | **84.7** | **84.2** | **80.8** | **88.0** |
| Bread | 47.9 | 44.0 | 50.6 | 54.2 | 48.9 | 60.1 |
| Ready-to-eat-cereals (RTEC) | 16.5* | 15.4* | 17.3 | 10.4* | 8.2* | 12.9 |
| Biscuits | 28.0 | 25.3 | 29.8 | 24.1 | 22.4 | 26.1 |
| Muffin | 10.4 | 11.7 | 9.5 | 9.8 | 11.6 | 7.8 |
| Cakes and pastries | 22.2 | 24.9* | 20.3 | 19.5 | 18.8* | 20.3 |
| Grains and flours | 9.7 | 9.9 | 9.5 | 7.1 | 5.6 | 8.7 |
| **Fruit + Juice** | **41.8*** | **42.9*** | **41.1** | **36.7*** | **34.7*** | **39.0** |
| Fresh fruit | 19.9* | 20.1* | 19.8 | 15.8* | 14.2* | 17.6 |
| Fruit nectar | 22.5* | 25.3* | 20.6* | 17.2* | 18.8* | 15.4* |
| Juices | 10.0 | 9.5 | 10.3 | 11.8 | 9.2 | 14.7 |
| Tomato | 26.2 | 30.0 | 23.6 | 26.4 | 28.1 | 24.5 |
| **Protein-rich foods** | **18.0** | **18.7** | **17.5** | **21.4** | **23.0** | **19.6** |
| Charcuterie and other meat products (chorizo, bacon, ham, sausages, etc) | 9.1* | 12.8* | 6.5 | 5.9* | 7.0* | 4.7 |
| Eggs | 1.9 | 2.2 | 1.8 | 1.2 | 1.6 | 0.7 |
| Meats | 1.9 | 2.2 | 1.8 | 1.9 | 1.8 | 2.0 |
| Fishes | 5.7 | 7.4 | 4.4 | 5.9 | 5.2 | 6.7 |
| **Other foods** | **4.9** | **6.6** | **3.8** | **4.5** | **3.8** | **5.3** |
| Greens and vegetables | 0.4 | 1.1 | 0 | 0.3 | 0.2 | 0.4 |
| Legumes | 0 | 0 | 0 | 0.2 | 0.2 | 0.2 |
| Pasta | 0.9 | 1.1 | 0.8 | 1.1 | 1.0 | 1.1 |
| Appetizers (crackers, chip potatoes, corn snacks, etc.) | 0.6 | 0.7 | 0.5 | 1.3 | 1.8 | 0.7 |
| Precooked foods (ready-to-eat creams and soups, croquettes, etc) | 9.4 | 6.0 | 11.8 | 7.6 | 6.0 | 9.3 |
| **Beverages** | **5.8** | **2.6** | **8.0** | **4.3** | **2.0** | **6.9** |
| Soya drinks | 1.9 | 2.2 | 1.8 | 1.7 | 2.4 | 0.9 |
| Sugared soft drinks | 1.2 | 1.1 | 1.3 | 0.6 | 0.2 | 1.1 |
| Sugar free soft drinks | 0 | 0 | 0 | 0.2 | 0.2 | 0.2 |
| Sports drinks | 0.3 | 0.4 | 0.3 | 0 | 0 | 0 |
| Other nonalcoholic beverages | 0.6 | 0 | 1.0 | 0.7 | 0.8 | 0.7 |
| Alcoholic beverages high alcohol (gin, whisky,etc) | 0 | 0 | 0 | 0.4 | 0.6 | 0.2 |
| Low-grade alcoholic beverages (wine, beer, cava, cider, etc) | 40.9 | 37.2 | 43.5 | 45.0 | 39.5 | 51.3 |
| **Fats** | **21.6** | **22.3** | **21.1** | **27.9** | **26.1** | **29.8** |
| Olive oil | 24.0 | 17.9 | 28.1 | 22.1 | 17.8 | 26.9 |
| Butter, margarine and other solid fats | 3.9 | 3.7 | 4.0 | 3.9 | 4.2 | 3.6 |
| Other oils | 86.8 | 84.0 | 88.7 | 89.9 | 86.2 | 94.0 |
| **Other products** | **60.9*** | **55.7*** | **64.4*** | **72.3*** | **67.7*** | **77.5*** |
| Cocoa | 27.4* | 30.0* | 25.6* | 20.1* | 21.6* | 18.5* |
| Sugars | 47.9 | 44.7 | 50.1 | 46.1 | 48.1 | 43.9 |
| Jams and others | 14.1 | 11.7 | 15.8 | 12.3 | 9.6 | 15.4 |
| Other chocolates | 11.5* | 12.8* | 10.5* | 5.4* | 6.4* | 4.2* |
| Saccharin | 8.2* | 6.6* | 9.3* | 16.7* | 12.0* | 22.0* |
| Sauces and condiments | 12.2 | 14.3 | 10.8 | 11.9 | 10.8 | 13.1 |
| Supplements and meal substitutes | 1.3 | 1.1 | 1.5 | 2.0 | 2.0 | 2.0 |

Z test proportions. The differences are between the same gender groups.* *p<*0.05
