# Supplementary material for: Correction: Breakfast habits and differences regarding abdominal obesity in a cross-sectional study in Spanish adults: The ANIBES study
Source: PLoS One. 2018 Aug 28;13(8):e0203341. doi: 10.1371/journal.pone.0203341 (PMC6112647; doi:10.1371/journal.pone.0203341)
Supplement: S2 Table — Differences regarding WHtR categories. Z test proportions. The differences are between the same gender groups.* p<0.05. (DOCX) [file pone.0203341.s001.docx]

**S2 Table. Foods consumed at breakfast (% consumers). Differences regarding WHtR categories.**

|  | **WHtR < 0.5** | | | **WHtR ≥ 0.5** | | |
| --- | --- | --- | --- | --- | --- | --- |
|  | **Total** | **Men** | **Women** | **Total** | **Men** | **Women** |
| **Dairy products** | **89.9** | **88.6** | **90.7** | **88.2** | **85.6** | **91.1** |
| Semi-skimmed cow milk | 43.5 | 42.5 | 44.1 | 44.1 | 42.7 | 45.7 |
| Whole cow milk | 30.7 | 36.3 | 26.8 | 26.5 | 29.5 | 23.2 |
| Skimmed cow milk | 19.2* | 15.8 | 21.6* | 24.4* | 21.2 | 28.1* |
| Other milks | 1.8 | 1.5 | 2.0 | 1.6 | 1.8 | 1.3 |
| Yoghurt and fermented milks | 8.8 | 12.5 | 6.3 | 7.7 | 8.8 | 6.5 |
| Cheese | 12.5 | 13.6 | 11.8 | 10.2 | 10.0 | 10.5 |
| Other dairy products (custard, curd, etc.) | 3.6 | 4.4 | 3.0 | 2.7 | 2.8 | 2.7 |
| **Cereals** | **84.1** | **83.2** | **84.7** | **84.2** | **80.8** | **88.0** |
| Bread | 47.9 | 44.0 | 50.6 | 54.2 | 48.9 | 60.1 |
| Ready-to-eat-cereals (RTEC) | 16.5* | 15.4* | 17.3 | 10.4* | 8.2* | 12.9 |
| Biscuits | 28.0 | 25.3 | 29.8 | 24.1 | 22.4 | 26.1 |
| Muffin | 10.4 | 11.7 | 9.5 | 9.8 | 11.6 | 7.8 |
| Cakes and pastries | 22.2 | 24.9* | 20.3 | 19.5 | 18.8* | 20.3 |
| Grains and flours | 9.7 | 9.9 | 9.5 | 7.1 | 5.6 | 8.7 |
| **Fruit + Juice** | **41.8*** | **42.9*** | **41.1** | **36.7*** | **34.7*** | **39.0** |
| Fresh fruit | 19.9* | 20.1* | 19.8 | 15.8* | 14.2* | 17.6 |
| Fruit nectar | 1.0 | 1.5 | 0.8 | 1.1 | 1.0 | 1.1 |
| Juices | 22.5* | 25.3* | 20.6* | 17.2* | 18.8* | 15.4* |
| Tomato | 10.0 | 9.5 | 10.3 | 11.8 | 9.2 | 14.7 |
| **Protein-rich foods** | **26.2** | **30.0** | **23.6** | **26.4** | **28.1** | **24.5** |
| Charcuterie and other meat products (chorizo, bacon, ham, sausages, etc) | 18.0 | 18.7 | 17.5 | 21.4 | 23.0 | 19.6 |
| Eggs | 9.1* | 12.8* | 6.5 | 5.9* | 7.0* | 4.7 |
| Meats | 1.9 | 2.2 | 1.8 | 1.2 | 1.6 | 0.7 |
| Fishes | 1.9 | 2.2 | 1.8 | 1.9 | 1.8 | 2.0 |
| **Other foods** | **5.7** | **7.4** | **4.4** | **5.9** | **5.2** | **6.7** |
| Greens and vegetables | 4.9 | 6.6 | 3.8 | 4.5 | 3.8 | 5.3 |
| Legumes | 0.4 | 1.1 | 0 | 0.3 | 0.2 | 0.4 |
| Pasta | 0 | 0 | 0 | 0.2 | 0.2 | 0.2 |
| Appetizers (crackers, chip potatoes, corn snacks, etc.) | 0.9 | 1.1 | 0.8 | 1.1 | 1.0 | 1.1 |
| Precooked foods (ready-to-eat creams and soups, croquettes, etc) | 0.6 | 0.7 | 0.5 | 1.3 | 1.8 | 0.7 |
| **Beverages** | **9.4** | **6.0** | **11.8** | **7.6** | **6.0** | **9.3** |
| Soya drinks | 5.8 | 2.6 | 8.0 | 4.3 | 2.0 | 6.9 |
| Sugared soft drinks | 1.9 | 2.2 | 1.8 | 1.7 | 2.4 | 0.9 |
| Sugar free soft drinks | 1.2 | 1.1 | 1.3 | 0.6 | 0.2 | 1.1 |
| Sports drinks | 0 | 0 | 0 | 0.2 | 0.2 | 0.2 |
| Other nonalcoholic beverages | 0.3 | 0.4 | 0.3 | 0 | 0 | 0 |
| Alcoholic beverages high alcohol (gin, whisky,etc) | 0.6 | 0 | 1.0 | 0.7 | 0.8 | 0.7 |
| Low-grade alcoholic beverages (wine, beer, cava, cider, etc) | 0 | 0 | 0 | 0.4 | 0.6 | 0.2 |
| **Fats** | **40.9** | **37.2** | **43.5** | **45.0** | **39.5** | **51.3** |
| Olive oil | 21.6 | 22.3 | 21.1 | 27.9 | 26.1 | 29.8 |
| Butter, margarine and other solid fats | 24.0 | 17.9 | 28.1 | 22.1 | 17.8 | 26.9 |
| Other oils | 3.9 | 3.7 | 4.0 | 3.9 | 4.2 | 3.6 |
| **Other products** | **86.8** | **84.0** | **88.7** | **89.9** | **86.2** | **94.0** |
| Coffee and tea | 60.9* | 55.7* | 64.4* | 72.3* | 67.7* | 77.5* |
| Cocoa | 27.4* | 30.0* | 25.6* | 20.1* | 21.6* | 18.5* |
| Sugars | 47.9 | 44.7 | 50.1 | 46.1 | 48.1 | 43.9 |
| Jams and others | 14.1 | 11.7 | 15.8 | 12.3 | 9.6 | 15.4 |
| Other chocolates | 11.5* | 12.8* | 10.5* | 5.4* | 6.4* | 4.2* |
| Saccharin | 8.2* | 6.6* | 9.3* | 16.7* | 12.0* | 22.0* |
| Sauces and condiments | 12.2 | 14.3 | 10.8 | 11.9 | 10.8 | 13.1 |
| Supplements and meal substitutes | 1.3 | 1.1 | 1.5 | 2.0 | 2.0 | 2.0 |

Z test proportions. The differences are between the same gender groups.* *p<*0.05
